# Who should be first? How and when AI-human order influences procedural justice in a multistage decision-making process

**DOI:** 10.1371/journal.pone.0284840

**Published:** 2023-07-17

**Authors:** Luyuan Jiang, Xin Qin, Kai Chi Yam, Xiaowei Dong, Wanqi Liao, Chen Chen

**Affiliations:** 1 Sun Yat-sen Business School, Sun Yat-sen University, Guangzhou, Guangdong, China; 2 Business School, National University of Singapore, Singapore, Singapore; University of Murcia: Universidad de Murcia, SPAIN

## Abstract

Artificial intelligence (AI) has fundamentally changed the way people live and has largely reshaped organizational decision-making processes. Particularly, AI decision making has become involved in almost every aspect of human resource management, including recruiting, selecting, motivating, and retaining employees. However, existing research only considers single-stage decision-making processes and overlooks more common multistage decision-making processes. Drawing upon person-environment fit theory and the algorithm reductionism perceptive, we explore how and when the order of decision makers (i.e., AI-human order vs. human-AI order) affects procedural justice in a multistage decision-making process involving AI and humans. We propose and found that individuals perceived a decision-making process arranged in human-AI order as having less AI ability-power fit (i.e., the fit between the abilities of AI and the power it is granted) than when the process was arranged in AI-human order, which led to less procedural justice. Furthermore, perceived AI ability buffered the indirect effect of the order of decision makers (i.e., AI-human order vs. human-AI order) on procedural justice via AI ability-power fit. Together, our findings suggest that the position of AI in collaborations with humans has profound impacts on individuals’ justice perceptions regarding their decision making.

## Introduction

In recent years, artificial intelligence (AI), which refers to “a highly capable and complex technology that aims to simulate human intelligence” [1], has been widely used to help make decisions in various areas, such as human resource management (HRM) [2, 3], tax management [4], sales management [5, 6], and customer service [7]. Particularly, given its high efficiency and performance [8–10], AI decision making has become involved in almost every aspect of HRM, including recruiting, selecting, motivating, and retaining employees, and has broad impacts on both employees and organizations [2, 3, 11, 12]. In this vein, one of the most important issues scholars have focused on is employees’ perceptions of the fairness of such decision-making procedures [13–15]. This is because fairness perceptions are strongly associated with employees’ attitudes and behaviors toward organizations [16, 17], and candidates are less likely to accept offers from organizations they deem to have a low level of organizational fairness [18–20]. Some emerging research also provided important insights about how AI characteristics (e.g., accuaracy, transparency) and individual difference (e.g., race, gender, education) affect fainess perceptions of AI decision making [21, 22]. However, several studies have shown that although AI has been demonstrated to have high efficiency and outperform human [23, 24], individuals tend to feel it is less fair when AI makes personnel decisions than when humans make personnel decisions, as they perceive that the AI decision-making process does not, or is unable to, consider the qualitive factors or unique social contexts from candidates [14, 25–27].

While previous studies provide the first look into the effects of AI on individuals’ fairness perceptions during decision-making processes, they mainly consider single-stage processes in which either AI or a human makes a decision independently, largely overlooking the complexity of managerial decision making in the real world (e.g., multistage decision-making processes). Indeed, in organizations, a final decision often unfolds in a multistage process [28–31], which involves a step-by-step approach whereby later available choices are based on early-stage decisions [28]. For example, top technology companies typically require at least five rounds of interview before an offer is made [32]. These include a series of interrelated stages, including initial screening interviews and later targeted interviews [33]. This process allows humans to make decisions in some stages, while AI makes decisions in other stages.

Although collaborations between humans and AI is ubiquitous in multistage organizational decision making [34–36], we have limited knowledge about the impacts of the order arrangement of AI and humans as decision makers on individuals’ fairness perceptions. In multistage decision-making, decision makers eliminate unacceptable alternatives and reduce the number of alternatives in the earlier stages and make the final choice from the remaining alternatives in the last stages [28, 31]. The order of decision makers (AI-human order vs. human-AI order) is a specific procedure design in AI-human joint decision-making context, reflecting who take charge to make decisions in the earlier stages and who are in the latest stage. Since individuals usually cannot immediately obtain the results of the personnel decision making, the order of decision makers provides direct information and impressions of decision-making process for individuals [37]. Thus, in this research, we focus on a typical two-stage personnel selection and explore (1) *whether* the order of decision makers (i.e., AI-human order vs. human-AI order) affects individuals’ procedural justice, which is the most intuitive perceptions and one of the fairness perceptions individuals care about most in the personnel decision-making [37], and (2) if so, *how and when* does this happen. Exploring these questions is theoretically important because doing so expands our knowledge of the neglected but ubiquitous phenomenon of multistage decision making involving both AI and humans in HRM. Practically, by understanding the influence of the order of decision makers (i.e., AI-human order vs. human-AI order), organizations could better design decision-making procedures when introducing AI to help make personnel decisions.

**Fig 1:**
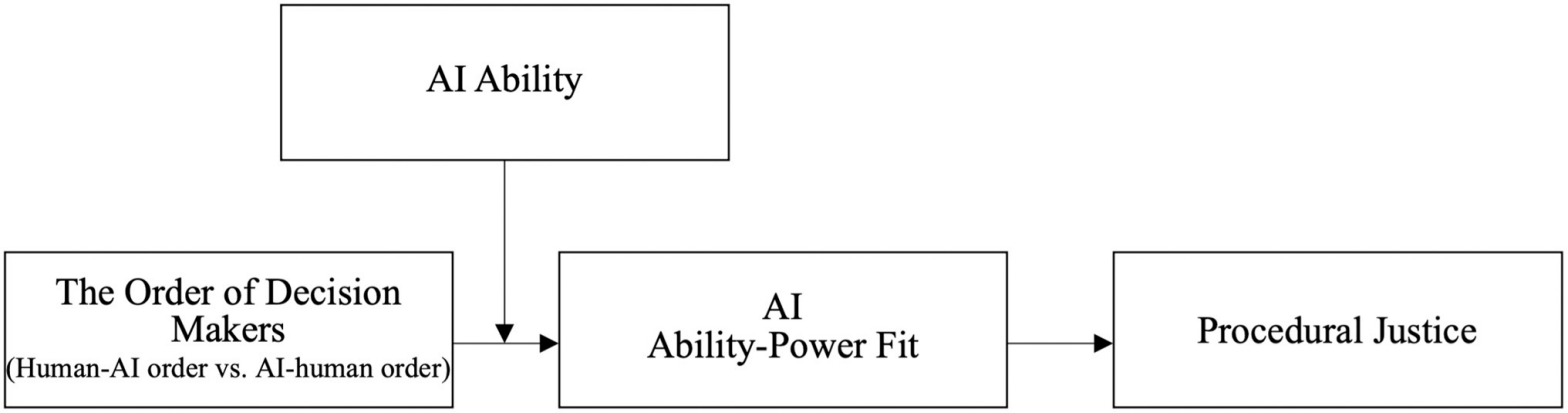
Theoretical model of the current research.

To answer these questions, we draw upon person-environment fit theory (P-E fit theory; [38, 39]) and the algorithmic reductionism perspective [14]. P-E fit theory suggests that outcomes are most optimal when the attributes of actors (e.g., the abilities of AI and humans) and the surrounding environment (e.g., the specific stage of the decision-making process) are compatible [38, 39], while the algorithmic reductionism perspective suggests that individuals tend to perceive that AI has lower levels of ability than humans due to its inability to consider certain qualitative information and contextualize this information [14]. Integrating P-E fit theory and the algorithmic reductionism perspective, we posit that in a multistage decision-making process, individuals perceive a lower level of AI ability-power fit (i.e., the fit between the abilities of AI and the power it is granted) and consequently decreased procedural justice (i.e, the perception of appropriateness in decision making procedures) when a human makes a decision in the first stage and AI makes a decision in the second stage (i.e., human-AI order condition) than in the reversed condition (i.e., AI-human order condition), since individuals tend to believe that decision makers in the second stage possess more power and, in turn, are required to have a higher level of ability compared with those in the first stage. Furthermore, as individuals’ perceptions of AI ability varies [40], we examine the moderating role of AI ability to provide a more complete understanding of when the order of decision makers has a stronger influence on procedural justice in a multistage decision-making process. To test our theoretical model (see Fig 1), we conducted two experimental studies.

This research makes several primary theoretical contributions. First, our research contributes to the AI decision-making literature by showing the importance of the order of decision makers (i.e., AI-human order vs. human-AI order) in a multistage decision-making process. Specifically, while previous studies typically consider a single-stage process by exploring how AI characteristics (e.g., accuaracy, transparency) and individual difference (e.g., race, gender, education) perceptionsof AI decision making, or comparing the decisions made by AI or a human independently [13–15, 41], this research considers a largely overlooked decision-making situation—that is, a multistage decision-making process—and explores effect of the order of decision makers (i.e., AI-human order vs. human-AI order), which is an important specific managerial design neglected in previous study, on individuals’ fairness perceptions. In doing so, this work expands our knowledge of individuals’ reactions in a multistage decision-making process that includes both AI and human decision makers. In addition, we identify an important boundary condition (i.e., AI ability), which helps us understand when the order of decision makers (i.e., AI-human order vs. human-AI order) has a stronger impact on procedural justice via AI ability-power fit.

Second, our research contributes to the literature on individuals’ fairness perceptions of AI decision making. Previous studies mostly consider how the attributes of AI (e.g., transparency, explainability, and accuracy) or the environment (e.g., task complexity) affect individuals’ fairness perceptions [42–46]. In this research, we suggest that individuals consider AI to be a “fit” for making decisions at a certain stage even when they perceive that AI has a lower level of ability than humans. Specifically, we reveal that individuals’ perceived procedural justice largely depends on congruence between AI ability and the power it is granted (i.e., AI ability-power fit).

## Theoretical grounding and hypothesis development

### Integrating person-environment fit and reductionism theories

P-E fit theory suggests that employees’ attitudes and behaviors are optimal when the attributes of actors and the surrounding environment are compatible [38, 39]. In the organizational context, P-E fit is often considered in terms of person-job fit, which refers to the fit between the attributes of a person and his or her job, including demands-abilities fit (i.e., the person’s abilities meet the requirements of the job he or she performs) and needs-supplies fit (i.e., the person’s needs are met by the job he or she performs) [18–20, 38, 47]. Moreover, job attributes can be conceptualized as different characteristics of a job, such as job autonomy, workload, job insecurity, role ambiguity, and (lack of) supervisor support [39].

Since we aim to explore how to arrange AI and humans to meet the requirements of a decision-making process at different stages, we conceptualize AI ability-power fit from the notion of abilities-demands fit and define it as the fit between the abilities of AI and the power it is granted. Since power is often viewed as a form of disproportionate control over other social actors [48–50], individuals tend to perceive that a position granted with more power has more control over their goals, which leads them to require the one who takes this position to have a higher level of ability so as to maximize their goal achievement [51–53]. Thus, when decision makers have a low level of ability in a position granted with a high level of power, they are more likely to be viewed as less capable of fulfilling the demands of such a position (i.e., misfit with this position) in the decision-making process. Taken into personnel selection, Gilliland suggests that a decision-making process should follow the 3 mian requirement: characteristics of the selection system, explanations offered during the selection process, and interpersonal treatment [54]. Thus, we proposed that AI ability-power reflects individuals’ perceptions on whether AI possess enough ability to follow appropriate way of evaluation (e.g., job relatedness), explanations offering (e.g., feedback), and interpersonal treatment (e.g., two-way communication) when making decisions at different positions.

Furthermore, to fully understand the effects of the order of decision makers in a multistage decision-making process, we integrate P-E fit theory [38, 39] with the algorithm reductionism perspective [14]. The algorithm reductionism perspective suggests that individuals tend to believe that AI does not have comparable abilities to make wise decisions as humans do because they think AI neglects “(1) the qualitative characteristics of human nature and, by extension, (2) the contextualized circumstances in which they occur” [14]. In a multistage decision-making process, the ability demands in different stages are quite different [55]. Thus, individuals may think it is a misfit when AI, rather humans, makes decisions at a specific stage granted with higher power.

### The order of decision makers and AI ability-power fit

In a multistage decision-making process, in particular a two-stage decision-making process, decision makers have different tasks and responsibilities at different stages: decision makers (1) eliminate unacceptable alternatives and reduce the number of alternatives in the first stage and (2) make the final choice from the remaining alternatives in the second stage [28, 31]. In the second stage, decision makers are typically granted more power and require higher levels of ability than those in the first stage, because the former may possess more power and thus more control over individual’s goal achievement. Specifically, decision makers’ power arises from the extent to which individuals’ goal achievement and gratification depend on them [49]. In terms of goal achievement in our context, an individual’s goal is to be accepted as the final personnel choice. The decision makers in the first stage could only partly affect an individual’s goal achievement by deciding who will pass the first selection round and move into the next round. While the decision makers in the second stage have more direct and critical impacts on the individual’s goal achievement by deciding who will be accepted as the final personnel choice [28, 56]. Thus, decision makers in the second stage have more control over individuals’ goal achievement and thus possess more power.

We posit that individuals perceive a lower level of AI ability-power fit when the decision-making process is arranged in human-AI order (vs. AI-human order). Specifically, when the decision-making process arranged in human-AI order (human-AI condition), the decision made by AI are considered to have more impact over interviewees’ success in personnel direction than decisions made by human, leading individuals perceive that AI possesses more power than human decision makers and consequently require AI to have higher ability than human to take the position to make decisions at the second stage. While the algorithm reductionism perspective [14] suggests that individuals tend to perceive that AI has a lower level of ability in making wise decisions than humans. Thus, individuals may view AI’s abilities as less likely to meet the requirements of the task (i.e., AI ability-power fit is low) in human-AI order condition. Conversely, individuals likely perceive a high level of AI ability-power fit when the decision-making process is arranged in AI-human order, since it meets their expectations that the decision makers (i.e., humans) in the second stage have a higher level of ability. Thus, we propose the following hypothesis:

*Hypothesis 1*: Individuals perceive a lower level of AI ability-power fit in the human-AI order condition than in the AI-human order condition.

### The effect of AI ability-power fit on procedural justice

Based on P-E fit theory [38, 39], we further propose that higher AI ability-power fit leads to higher perceptions of procedural justice, reflecting individuals perceptions of the appropriateness in decision-making procedures [57, 58], which is the most intuitive perceptions and one of the fairness perceptions individuals care about most in the personnel decision-making [37]. Gilliland suggests that individuals may feel lower procedural justice when the rules of a decision-making process are violated in three dimensions: characteristics of the selection system (e.g., job relatedness), explanations offered during the selection process (e.g., feedback), and interpersonal treatment (e.g., two-way communication) [54]. Since these three dimensions can greatly affect the individual’s perception of procedural justice, decision makers should have the ability to match their power (i.e., ability-power fit). Specifically, the above three aspects form the backbone of the abilities that decision makers should have.

Based on Gilliland’s theoretical model [54], we suggest that AI ability-power fit is positively related to procedural justice through these three elements. Specifically, first, when individuals perceive a low level of AI ability-power fit, they may think that AI does not have the abilities to accurately measure the content relevant to the job situation as required by its position in the decision-making process, which is a violation of the accuracy rule of formal characteristics [54, 59, 60]. Second, when individuals perceive a low level of AI ability-power fit, they may believe AI cannot give timely and informative feedback when making decisions, which violates the rule of explanation [54, 61]. Third, they may also consider that the social skills required for decision making are beyond AI’s abilities and will thus lead to limited communication, which is important for interpersonal treatment [37, 54, 59]. Accordingly, when individuals perceive a low level of AI ability-power fit, they may believe that AI does not have the abilities to make accurate decisions at its position, to give timely and informative feedback, and to enable two-way communication, thus leading these individuals to think the decision-making process is less fair [54]. Combining these arguments with Hypothesis 1, we propose the following hypotheses:

*Hypothesis 2*: AI ability-power fit is positively related to procedural justice.

*Hypothesis 3*: The order of decision makers (i.e., AI-human order vs. human-AI order) has an indirect effect on procedural justice via AI ability-power fit.

### The moderating role of AI ability

Thus far, we have proposed the indirect effect of the order of decision makers (i.e., AI-human order vs. human-AI order) on procedural justice via AI ability-power fit. We further argue that AI ability is an important boundary condition for this relationship. Based on P-E fit theory [38, 39] and the algorithm reductionism perspective [14], when a human is the decision maker in the first stage and AI is the decision maker in the second stage, individuals tend to feel a lower level of AI ability-power fit since people tend to believe AI’s abilities are lower than a human’s abilities. Thus, we suggest that this relationship is buffered when AI ability is high.

Specifically, when AI ability is low, individuals are more likely to consider AI as less efficient and lower quality, which means they tend to perceive a greater gap between the abilities of AI and humans. Consequently, individuals are more likely to think that only humans, rather than AI, can meet the requirements of the second stage in the two-stage decision-making process we discussed above. Thus, when AI ability is low, individuals likely tend to feel a lower level of AI ability-power fit in the human-AI order condition than in the AI-human order condition. However, when AI ability is high, individuals may perceive a smaller gap between the abilities of AI and humans and may even believe that both AI and humans are capable of being decision makers in the second stage. As such, they likely tend to feel there are only small differences in the decision-making process in the human-AI order condition versus the AI-human order condition. Combining this rationale with Hypothesis 3, we further suggest that perceived AI ability is an important boundary condition under which the order of decision makers (i.e., AI-human order vs. human-AI order) has a stronger or weaker impact on individuals’ procedural justice perception. In sum, we propose the following hypotheses:

*Hypothesis 4*: AI ability moderates the relationship between the order of decision makers (i.e., AI-human order vs. human-AI order) and AI ability-power fit such that individuals perceive a lower level of AI ability-power fit in the human-AI order condition than in the AI-human order condition when AI ability is low (vs. high).

*Hypothesis 5*: AI ability moderates the indirect effect of the order of decision makers (i.e., AI-human order vs. human-AI order) on procedural justice via AI ability-power fit such that individuals perceive a lower level of procedural justice in the human-AI order condition than in the AI-human order condition via AI ability-power fit when AI ability is low (vs. high).

### Overview of current research

To test our theoretical model, we conducted two experimental studies. In Study 1, we manipulated the order of decision makers (i.e., AI-human order vs. human-AI order) and examined the indirect effect of the order of decision makers on procedural justice via AI ability-power fit in an interview decision-making context. In Study 2, we manipulated the order of decision makers and examined the indirect effect of the order of decision makers on procedural justice via AI ability-power fit in a promotion decision-making context. In addition, we manipulated AI ability and tested the entire theoretical model in Study 2. Full data of all studies can be found online at https://osf.io/bu67v/?view_only=e4e3ace663674982aa71f8bfc455377b.

## Study 1 method

### Participants

We recruited 134 employees from the United States via Prolific, a widely used online survey platform [62–64]. In both studies, we complied with the ethical standards of the Declaration of Helsinki and common institutional review board (IRB) regarding data collection procedures, even though the Chinese institutions that employ the authors in charge of data collection did not have an IRB. In particular, participants’ confidentiality was guaranteed throughout the entire data collection processes. We obtained a written participant consent from human subjects in the experiment, where they were asked whether they would like to participate and were allowed to withdraw from the study at any given time. This sample size ensured a power level of 0.80 to detect a medium effect (*f* = 0.25), assuming an *α* level of 0.05 [65]. Each participant in this research received 0.5 USD as compensation. Among them, 78.4% were female and 81.3% were White. Their average age was 29.4 years old (*SD* = 8.3), average education was 16.5 years (*SD* = 3.0), and average organizational tenure was 4.0 years (*SD* = 3.9). Participants worked in a variety of industries, including healthcare (18.7%), education (14.2%), information technology (12.7%), service (8.1%), and others (46.3%). They were also from different departments, including technology related (23.9%), administration related (23.9%), finance related (4.9%), marketing related (4.9%), and others (43.3%).

### Procedure and experiment design

We manipulated the order of decision makers (i.e., interviewers) in an employee-hiring decision-making context, resulting in two conditions (i.e., AI-human order vs. human-AI order). We randomly assigned participants to one of the two scenarios: a two-round interview process arranged in either AI-human order (*n* = 67) or human-AI order (*n* = 67). After reading the scenario, participants completed measurements of AI ability-power fit and procedural justice, completed a manipulation check, and reported their demographic information.

#### Manipulation of the order of decision makers

We manipulated the order of decision makers by instructing participants to read a statement describing a two-round interview process including both AI and human interviewers (for similar research designs, see Longoni et al.’s [66] and Newman et al.’s [14] research). Each scenario began with the following background of the employee-hiring interview context:

*Company X is switching from a traditional interview process for employee hiring to including an artificial intelligence (AI) interview*. *Specifically*, *in the interview procedure of Company X*, *there are two rounds of interviews*.

Then, we presented the two-round design of the interview process to manipulate the order of decision makers. Specifically, in the AI-human order condition, participants read the following statement:

*In the first round*, *AI interviewers (i*.*e*., *algorithm-based decision-making agents) will interview applicants and decide which applicants will pass this interview round*. *In the second round*, *human interviewers (i*.*e*., *the company’s division managers) will further interview applicants and decide which applicants will get offers*.

In the human-AI order condition, participants read another statement:

*In the first round*, *human interviewers (i*.*e*., *the company’s division managers) will interview applicants and decide which applicants will pass this interview round*. *In the second round*, *AI interviewers (i*.*e*., *algorithm-based decision-making agents) will further interview applicants and decide which applicants will get offers*.

### Measures

Unless otherwise specified, all measures for the two studies used a five-point Likert-type format ranging from 1 = “*Strongly disagree*” to 5 = “*Strongly agree*.” All items are available in S1 Appendix.

#### Perceived AI ability-power fit

We measured perceived AI ability-power fit using a four-item scale adapted from Cable and DeRue’s person-job fit scale [67]. A sample item is “The match is very good between AI’s abilities and the power it has been granted in this interview process” (*α* = .96).

#### Procedural justice

We measured procedural justice using Newman et al.’s four-item scale [14], which was adapted from Conlon et al.’s research [68]. A sample item is “The way this interview process determines which candidates receive job opportunities seems fair” (*α* = .95).

#### Manipulation check

We asked participants to recall and rate the extent to which they agreed with two statements about the order of the interviewers in the scenario they read. The two items are “In the two interview rounds presented in the scenario above, AI interviewers (rather than humans) are the first-round interviewers” and “In the two interview rounds presented in the scenario above, humans (rather than AI interviewers) are the second-round interviewers” (*α* = .93).

### Analytic strategy

To test our hypotheses, we conducted two-sample *t*-tests by condition and ordinary least squares (OLS) regressions. To test the mediating effect, we employed the PROCESS macro (v3.5; Model 4) [69] to estimate the indirect effect of the order of decision makers on procedural justice via AI ability-power fit using 5,000 bootstrapped resamples.

## Study 1 results and discussion

### Manipulation check

Participants in the AI-human condition agreed that the decision-making process was arranged in AI-human order (*M* = 4.82, *SD* = .54) significantly more than those in the human-AI order condition (*M* = 1.32, *SD* = .88), *t*(132) = 27.68, *p* < .001, *d* = 4.78. These results suggested that our manipulation was successful.

### Tests of hypotheses

The descriptive statistics and correlations are presented in Table 1.

**Table 1:** Descriptive statistics and correlation coefficients of variables in Study 1.

Variables	Mean	*SD*	1	2
1. The order of decision makers	.50	.50		
2. AI ability-power fit	2.88	1.00	-.25**	
3. Procedural justice	3.04	.98	-.24**	.85***

*Note*. *n* = 134. For the order of decision makers, AI-human condition = 0, human-AI condition = 1.

*p < .05,

** *p* < .01,

***p < .001.

Hypothesis 1 proposes that individuals perceive a lower level of AI ability-power fit in the human-AI order condition than in the AI-human order condition. The two-sample *t*-test results showed that participants perceived a higher level of AI ability-power fit in the AI-human order condition (*M* = 3.13, *SD* = .97) than in the human-AI order condition (*M* = 2.63, *SD* = .96), *t*(132) = 2.97, *p* = .004, *d* = .51. The result of OLS regression also showed that the order of decision makers had a significant and negative relationship with AI ability-power fit (Model 1, Table 2; *b* = -.50, *p* = .004). Thus, Hypothesis 1 was supported.

**Table 2:** The main effect of the order of decision makers on procedural justice in Study 1.

Variables	AI ability-power fit			Procedural justice					
	Model 1			Model 2			Model 3		
	*b*	*SE*	*t*	*b*	*SE*	*t*	*b*	*SE*	*t*
The order of decision makers	-.50	.17	-2.96**	-.47	.17	-2.87**	-.06	.10	-0.66
AI ability-power fit							.83	.05	17.94***
Constant	3.13	.12	26.36***	3.28	.12	28.03***	.68	.16	4.30***
*R* ^*2*^	.06**			.06**			.73***		

*Note*. *n* = 134; *n* = 67 in the AI-human condition; *n* = 67 in the human-AI condition. For the order of decision makers, AI-human condition = 0, human-AI condition = 1.

*p < .05,

** *p* < .01,

***p < .001.

Hypothesis 2 proposes that AI ability-power fit is positively related to procedural justice. As shown in Model 3 of Table 2, AI ability-power was positively related to procedural justice (*b* = .83, *p* < .001). Thus, Hypothesis 2 was supported.

Hypothesis 3 proposes that the order of decision makers has an indirect effect on procedural justice via AI ability-power fit. The two-sample *t*-test result showed that participants perceived a higher level of procedural justice in the AI-human order condition (*M* = 3.28, *SD* = .96) than in the human-AI order condition (*M* = 2.80, *SD* = .95), *t*(132) = 2.87, *p* = .005, *d* = .50. The result of OLS regression also showed that the order of decision makers had a significant and negative relationship with procedural justice (Model 2, Table 2; *b* = -.47, *p* = .005). The bootstrapping results revealed a significant indirect effect of the order of decision makers on procedural justice via AI ability-power fit (estimate = -.41, *SE* = .14, 95% CI = [-.69, -.14]). Thus, Hypothesis 3 was supported.

These results offered initial support for Hypotheses 1, 2, and 3. Study 1 demonstrated that the order of decision makers (i.e., AI-human order vs. human-AI order) affects procedural justice via AI ability-power fit. Specifically, compared with interviews arranged in AI-human order, individuals in interviews arranged in human-AI order perceive less AI ability-power fit, which decreases their procedural justice. Although we conducted this study in a typical multistage decision-making process—namely, for hiring decisions—we did not know whether our findings would hold in other situations. Therefore, we conducted Study 2 to extend our findings to a promotion decision context. In addition, we included the moderator (i.e., AI ability) to test our entire theoretical model in Study 2.

## Study 2 method

### Participants

We recruited 183 full-time employees from China via the authors’ alumni networks. As in Study 1, informed consent was obtained from all participants. This sample size ensured a power level of 0.80 to detect a medium effect size (*f* = 0.25), assuming an *α* level of 0.05 for our 2 x 2 factorial design [65]. Each participant in this research received 0.5 USD as compensation. Among them, 59.6% were female. Their average age was 29.1 years old (*SD* = 6.6), average education was 16.9 years (*SD* = 1.7), and average organizational tenure was 4.0 years (*SD* = 5.3). Participants worked in a variety of industries, including banking (25.7%), information technology (12.0%), education (11.5%), manufacturing (10.9%), and others (39.9%). Participants were from different departments, including technology related (27.9%), administration related (19.1%), finance related (16.4%), marketing related (10.4%), and others (26.2%).

### Procedure and experiment design

Using a 2 (the order of decision makers: human-AI order vs. AI-human order) × 2 (AI ability: low vs. high) factorial design, we manipulated both the order of decision makers and AI ability in a promotion decision context. We randomly assigned participants to one of these four conditions. To manipulate the order of decision makers, we adapted the scenario in Study 1 to a promotion decision context. To manipulate AI ability, we gave participants detailed information about the abilities of the AI decision makers based on the experimental scenarios of Langer et al. [70] and Newman et al. [14]. In line with Study 1, participants then completed measurements of AI ability-power fit, procedural justice, and manipulation checks, and reported their demographic information.

#### Manipulation of AI ability

We first instructed participants to read the background information of a two-round promotion decision process, which was adapted from Study 1. After that, to manipulate AI ability, we supplied detailed information about the abilities of the AI used in this process. Specifically, in the high AI ability condition, participants were asked to read the following:

*Company X is switching from a traditional decision-making process for employee promotion to including artificial intelligence (AI) agents*. *The AI agents that Company X currently employs can recognize the abilities and personal qualities of candidates*, *can evaluate candidates systematically*, *and rarely miss information related to future performance*.

In the low AI ability condition, participants were asked to read the following:

*Company X is switching from a traditional decision-making process for employee promotion to including artificial intelligence (AI) agents*. *The AI agents that Company X currently employs cannot fully recognize the abilities and personal qualities of candidates*, *can evaluate candidates on only a few aspects*, *and may miss information related to future performance*.

#### Manipulation of the order of decision makers

Adapted from Study 1, we presented a two-round promotion decision scenario to manipulate the order of decision makers. Specifically, in the AI-human order condition, participants read the following statement:

*Specifically*, *in the decision-making process of Company X*, *there are two rounds of assessments*. *In the first round*, *AI (i*.*e*., *algorithm-based decision-making agents) will evaluate candidates and decide which candidates will pass this assessment round*. *In the second round*, *humans (i*.*e*., *the company’s division managers) will further evaluate candidates and decide which candidates will get promoted*.

In the human-AI order condition, participants read another statement:

*Specifically*, *in the decision-making process of Company X*, *there are two rounds of assessments*. *In the first round*, *humans (i*.*e*., *the company’s division managers) will evaluate candidates and decide which candidates will pass this assessment round*.

*In the second round*, *AI (i*.*e*., *algorithm-based decision-making agents) will further evaluate candidates and decide which candidates will get promoted*.

### Measures

All scales in Study 2 were translated into Mandarin Chinese following a translation-back translation procedure based on Brislin’s suggestions [71].

#### Perceived AI ability-power fit

We measured perceived AI ability-power fit using the same scale in Study 1 (*α* = .90).

#### Procedural justice

We measured procedural justice using the same scale in Study 1 (*α* = .92).

#### Manipulation check

Similar to Study 1, we asked participants to recall and rate the extent to which they agreed with two statements about the order of decision makers in the scenario they read. A sample item is “In the two rounds of promotion presented in the scenario above, AI interviewers (rather than humans) are the first round decision makers” (*α* = .93). To check the manipulation of AI ability, we asked participants to rate the abilities of AI in the presented scenario using a three-item scale adapted from Newman et al. [14]. A sample item is “In this promotion decision-making process, AI could put all factors related to future performance into context when evaluating candidates” (*α* = .78).

### Analytic strategy

In line with Study 1, we conducted two-sample *t*-tests and OLS regressions to test our hypotheses and estimated the indirect effects using the PROCESS macro (Model 4). To test the moderated mediation effect, we conducted bootstrapping-based mediation analyses using the PROCESS macro (Model 7) with 5,000 bootstrapped resamples and estimated the conditional indirect effects at high and low levels of the moderator [72, 73].

## Study 2 results

### Manipulation check

Regarding the manipulation check for the order of decision makers, participants in the AI-human condition agreed that the decision-making process was arranged in AI-human order (*M* = 4.09, *SD* = .68) significantly more than those in the human-AI condition (*M* = 2.46, *SD* = 1.36), *t*(181) = 10.28, *p* < .001, *d* = 1.52. Regarding the manipulation check for AI ability, participants in the high AI ability condition rated the AI as having a higher level of ability (*M* = 3.33, *SD* = .73) than those in the low AI ability condition (*M* = 2.99, *SD* = .89), *t*(181) = -2.83, *p* = .005, *d* = .42. These results suggested that our manipulations were successful.

### Tests of hypotheses

Table 3 shows the descriptive statistics and correlations.

**Table 3:** Descriptive statistics and correlation coefficients of variables in Study 2.

Variables	Mean	*SD*	1	2	3
1. The order of decision makers	.49	.50			
2. AI ability	.50	.50	-.01		
3. AI ability-power fit	3.24	.88	-.33***	.15*	
4. Procedural justice perceptions	3.31	.84	-.29***	.11	.72***

*Note*. *n* = 183. For the order of decision makers, AI-human condition = 0, human-AI condition = 1. For AI ability, low AI ability condition = 0, high AI ability condition = 1.

*p < .05,

** *p* < .01,

***p < .001.

Hypothesis 1 proposes that individuals perceive a lower level of AI ability-power fit in the human-AI order condition than in the AI-human order condition. The two-sample *t*-test results showed that participants in the human-AI condition perceived a lower level of AI ability-power fit (*M* = 2.94, *SD* = .96) than those in the AI-human condition (*M* = 3.52, *SD* = .70), *t*(181) = 4.63, *p* < .001, *d* = .68. The result of OLS regression also showed that the order of decision makers had a significant and negative relationship with AI ability-power fit (Model 1, Table 4; *b* = -.57, *p* < .001). Thus, Hypothesis 1 was supported.

**Table 4:** The main and interactive effects of the order of decision makers and AI ability on procedural justice in Study 2.

Variables	AI ability-power fit						Procedural justice								
	Model 1			Model 2			Model 3			Model 4			Model 5		
	*b*	*SE*	*t*	*b*	*SE*	*t*	*b*	*SE*	*t*	*b*	*SE*	*t*	*b*	*SE*	*t*
The order of decision makers	-.57	.12	-4.67***	-.95	.17	-5.67***	-.48	.12	-4.02***	-.10	.09	-1.07	-.18	.13	-1.36
AI ability	.27	.12	2.19*	-.11	.17	-0.67	.18	.12	1.50	.00	.09	.01	-.07	.12	-0.59
The order of decision makers × AI ability				.77	.24	3.24**							.16	.18	.86
AI ability-power fit										.67	.05	12.55***	.66	.05	11.99***
Constant	3.38	.11	32.01***	3.57	.12	30.09***	3.45	.10	33.44***	1.19	.20	6.10***	1.27	.21	5.91***
*R* ^*2*^	0.13***			0.18***			0.09**			0.52***			0.52***		

*Note*. *n* = 183; *n* = 47 in the AI-human order and high AI ability condition; *n* = 46 in the AI-human order and low AI ability condition; *n* = 45 in the human-AI order and high AI ability condition; *n* = 45 in the human-AI order and low AI ability condition. For the order of decision makers, AI-human condition = 0, human-AI condition = 1. For AI ability, low AI ability condition = 0, high AI ability condition = 1.

*p < .05,

** *p* < .01,

***p < .001.

Hypothesis 2 proposes that AI ability-power fit is positively related to procedural justice, while Hypothesis 3 proposes that the order of decision makers has an indirect effect on procedural justice via AI ability-power fit. As shown in Table 4, the results showed that AI ability-power fit was positively related to procedural justice (*b* = .67, *p* < .001). The two-sample *t*-test results showed that participants perceived a lower level of procedural justice in the human-AI order condition (*M* = 3.06, *SD* = .61) than those in the AI-human order condition (*M* = 3.55, *SD* = .98), *t*(181) = 4.02, *p* < .001, *d* = .60. The results of the OLS regressions also showed that the order of decision makers had a significant and negative relationship with procedural justice (Model 3, Table 4; *b* = -.48, *p* < .001). As shown in Table 5, the order of decision makers had a significant indirect effect on procedural justice via AI ability-power fit (estimate = -.38., *SE* = .10, 95% CI = [-.59, -.21]). Thus, Hypotheses 2 and 3 were supported.

**Table 5:** Analyses of conditional indirect effects in Study 2.

Paths and Effects	Estimate	SE	95% confidence intervals
**The order of decision makers → AI ability-power fit → Procedural justice**			
Simple indirect effect	-.38	.10	[-.59, -.21]
Moderated mediation			
Lower AI ability	-.64	.15	[-.95, -.37]
Higher AI ability	-.13	.11	[-.34, .08]
Difference	.52	.17	[.19, .87]

*Note*. *n* = 183.

**Fig 2:**
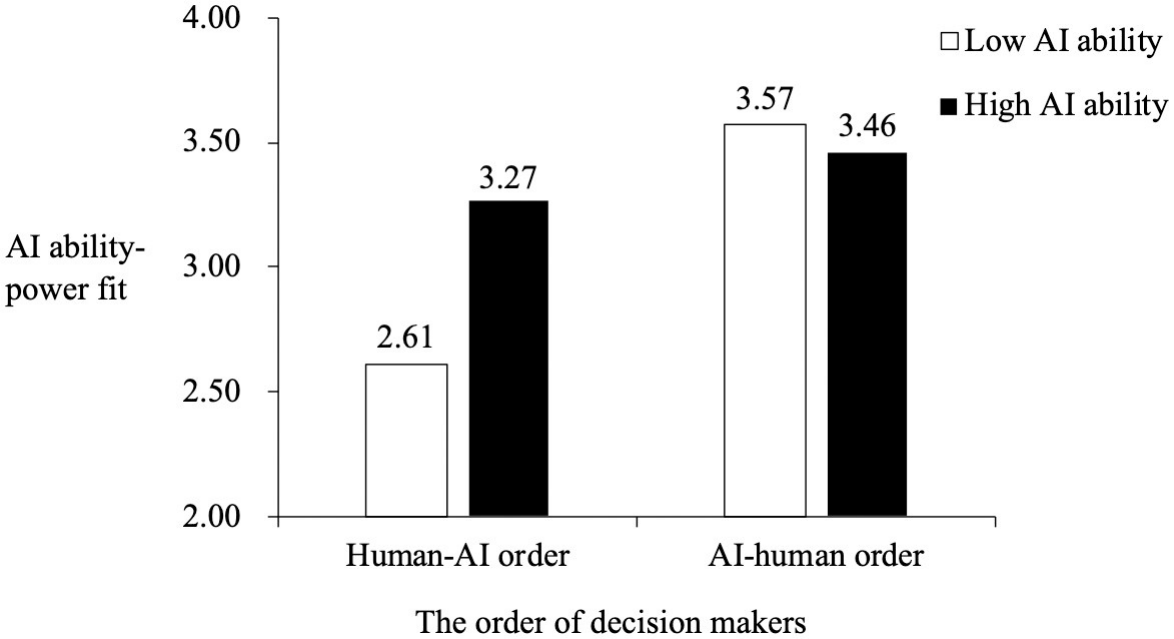
The moderating effect of AI ability.

To test Hypotheses 4 and 5, we used the PROCESS macro (Model 7) to test the moderated mediation model. As shown in Table 4, the interaction effect of the order of decision makers (i.e., AI-human order vs. human-AI order) and AI ability in predicting AI ability-power fit was significant (*b* = .77, *p* = .001). As depicted in Fig 2, simple slope tests indicated that the relationship between the order of decision makers and AI ability-power fit was significant and negative when AI ability was low (*b* = -.96, *p* < .001) but was not significant when AI ability was high (*b* = -.19, *p* = .25). Furthermore, the results in Table 5 revealed that the indirect effect of the order of decision makers on procedural justice via AI ability-power fit was significant when AI ability was low (estimate = -.64, *SE* = .15, 95% CI = [-.95, -.37]) but was not significant when AI ability was high (estimate = -.13, *SE* = .11, 95% CI = [-.34, .08]). The difference between these indirect effects was also significant (*Δ*estimate = .52, *SE* = .17, 95% CI = [.19, .87]). Thus, Hypotheses 4 and 5 were supported.

## General discussion

Previous research has typically compared humans and AI in making single-stage decisions [14, 74]. However, in organizations, a final decision often unfolds in a multistage process [28–31]. Drawing upon P-E fit theory [38, 39] and the algorithm reductionism perspective [14], we focus on two-stage personnel decision-making processes and propose how and when the order of decision makers (i.e., AI-human order vs. human-AI order) affects individuals’ perceptions of procedural justice. Across two experimental studies, we found that, compared to those in the AI-human order condition, individuals in the human-AI order condition perceived a lower level of AI ability-power fit, which led to a lower level of perceived procedural justice. Furthermore, AI ability attenuated the effect of the order of decision makers on procedural justice via AI ability-power fit.

### Implications for theory

Our study makes several important theoretical contributions to the literature on AI decision making by exploring a multistage decision-making process. First, previous studies only explored how AI characteristics (e.g., accuaracy, transparency) and individual difference (e.g., race, gender, education) affect perceptionsof AI decision making, or compared the decisions made by AI or a human, which are largely based on situations in which AI makes decisions independently [14, 41, 75, 76]. However, in organizations, a final decision often unfolds in a multistage decision-making process involving both AI and humans [30, 31, 77]. This research explores an specific managerial design in AI decision-making process by identifying an important antecedent—the order of decision makers (i.e., AI-human order vs. human-AI order) on individuals’ justice perceptions AI decision-making process. By doing so, our study contributes to the AI decision-making literature by considering a neglected but ubiquitous context—multistage decision making—which is more common in actual workplaces and thus enables organizations to better understand the impacts of AI decision making. Furthermore, our study also provides some insights into human and AI jointly decision-making by considering an specific managerial design in personnel selection.

Second, our study contributes to the literature on AI fairness perceptions by exploring an important but neglected antecedent—AI ability-power fit. Our study emphasizes that the perceived fairness of AI is affected by the congruence between the attributes of AI (i.e., AI’s abilities) and its position (i.e., different stages in a decision-making process). However, existing research on AI fairness perceptions views the effects of AI characteristics (e.g., transparency, explainability, accuracy) and environmental characteristics (e.g., task complexity) on AI fairness perceptions separately [42–46]. Based on P-E theory [38, 39] and the algorithm reductionism perspective [14], in this research, we found that AI ability-power fit was positively related to procedural justice. Thus, our research contributes to the literature on AI fairness perceptions by arguing that individuals’ fairness perceptions are determined not only by AI or the environment separately, but also by their fit or congruence with each other (i.e., AI ability-power fit). We also found that AI ability is a boundary condition such that AI ability-power fit and procedural justice are much lower in the human-AI order condition than in the AI-human order condition when AI ability is low (vs. high).

Third, our study contributes to P-E theory [38] by exploring an emerging social actor—AI. Indeed, existing research on P-E fit provides deep insights into the fit between people and their surrounding environments, such as person-organization fit, person-job fit, person-group fit, and person-supervisor fit [18, 78]. Since AI has become an important social actor in organizations [5, 79], our study extends P-E fit theory by exploring the fit between AI’s abilities and the power it is granted (i.e., AI ability-power fit). In particular, our study shows that when a two-stage decision-making process is arranged in human-AI order (vs. AI-human order), individuals tend to perceive a lower level of AI ability-power fit because they perceive that AI’s abilities is lower than humans’ abilities and that AI is not competent enough to match the power needed to make final decisions in the second stage. This finding provides some insights regarding the fit between AI and its surrounding environments. In sum, our study extends P-E fit theory by viewing AI as a social actor and exploring the fit between AI’s abilities and the power it is granted, which is important for understanding the phenomenon of AI becoming an increasingly important decision maker in HRM.

### Implications for practice

Our findings also provide several important managerial implications. First, our work may provide important guidelines for practitioners to understand the position of AI in multistage decision making. We found that the decision-making process arranged in human-AI order (vs. human-AI order) led individuals to perceive lower AI ability-power fit and consequently less procedural justice. Although previous studies suggest there is a problem with AI making personnel-selection decisions (i.e., lack of perceived fairness), our findings show that an appropriately arranged decision-making process—namely, when AI makes decisions in the first stage and humans make decisions in the second stage—may help reduce this potential cost in multistage decision-making processes. That is, AI should be located in the earlier stage(s) of a decision-making process while humans should be located in the later stage(s).

Second, we recommend managers to reconsider AI’s roles and positions discreetly so as to ensure that AI’s abilities and power are matched reasonably in collaborations with humans. Our findings reveal that individuals’ attitudes (i.e., fairness perceptions) are largely affected by the compatibility of the attributes of AI and humans (i.e., abilities of AI and humans) and the respective tasks and responsibilities they take on. Thus, managers should be cautious when placing AI in higher positions than human decision makers due to the possible mismatch between AI’s abilities and the power it is granted.

Third, our findings help organizations make full use of both high-ability AI and low-ability AI. Since our findings indicate that AI ability moderates the effect of the order of decision makers on individuals’ perceived procedural justice, we come to realize AI with both high and low ability has its own use. In particular, we suggest that when AI ability is low, AI is a suitable choice for initial screening and assisting human decision makers. When AI ability is high, AI could also take charge of some important tasks in collaborations with humans. Thus, we encourage managers to consider the differences and advantages of the two types of AI, which means not blindly chasing AI with the best ability nor having too much confidence in low-ability AI.

### Strengths, limitations, and future directions

While our research has a variety of strengths (e.g., multi-studies conducted in both America and China), there are some limitations that should be discussed and addressed by future research. First, although we conducted two scenario-based experiments in different context to enhance external validity of our foundings, our study still some limited in taking account the richeness of real organizational settings. In other words, all our finding are based on simulated decision-making process in personnel selection, and individuals might have different feelings when they are actually seeking for a job. Thus, we encourage future research to conduct field experiments and surveys to replicate these findings. For example, future studies can collaborat with companies that apply AI to personnel selection and randomly placing applicants into AI-human order condition or human-AI order condition in recruitment activities to explore their fairness perceptions, thus examining and extending our findings in actual organizational settings. In addition, in order to make it easy to understand for participants, we used the general scale to measure AI ability-power fit. Future research might also develop use specific AI ability-power fit scale that capture the specific abilities required in the actual field experiment.

Second, while we tested our hypotheses in a typical two-stage decision-making process, there are more complicated decision-making processes in actual organizational settings [80, 81]. For example, in personnel selection, individuals also have to receive an evaluation at an assessment center after their initial screening interview and later target interview [82, 83]. Furthermore, not all decision tasks are the same at each stage. Thus, we encourage future research to explore such decision-making processes that are more than two stages and more than one kind of task.

Third, our research only focused on the perceptions of applicants. However, human resource decision making involves other important actors, such as HR specialists and division managers. Future research can examine whether the order of decision makers (i.e., AI-human order vs. human-AI order) affects HR specialists’ or division managers’ perceptions and behaviors. For example, HR specialists may perceive less respect when they make decisions in the first stage and AI makes final decisions because this order may convey that organizations affirm the value of AI and deny their contributions, which may consequently lead to less organizational commitment in such employees [84]. Thus, it would be promising for future research to pay attention to other relevant actors and explore how AI decision making affects their perceptions and behaviors.

## Conclusion

This research concentrated on a neglected but ubiquitous context—a multistage decision-making process involving AI and humans. Drawing upon P-E fit theory [38, 39] and the algorithm reductionism perspective [14], we propose and found that a decision-making process arranged in human-AI order (vs. AI-human order) leads individuals to perceive less AI ability-power fit and procedural justice, especially when AI ability is low. By identifying the roles and positions of AI in collaborations with humans, we hope our findings encourage future scholars to reconsider AI decision making in more complex and dynamic social environments.

## Supporting information

S1 Appendix Scale items used in Studies 1 and 2. (DOCX)
