# Correction: Who should be first? How and when AI-human order influences procedural justice in a multistage decision-making process

**DOI:** 10.1371/journal.pone.0334830

**Published:** 2025-10-16

**Authors:** Luyuan Jiang, Xin Qin, Kai Chi Yam, Xiaowei Dong, Wanqi Liao, Chen Chen

The authors, Luyuan Jiang, Xin Qin and Chen Chen, should not have been attributed equal contribution to this work.

The authors, Kai Chi Yam, Xiaowei Dong and Wanqi Liao, should not have been attributed equal contribution to this work.

The affiliation for the first author is incorrect. The correct affiliation is not indicated. Luyuan Jiang is not affiliated with #1 but with: School of Economics and Management, China University of Mining and Technology, Xuzhou, Jiangsu, China.
