# Supplementary material for: Who should be first? How and when AI-human order influences procedural justice in a multistage decision-making process
Source: PLoS One. 2023 Jul 17;18(7):e0284840. doi: 10.1371/journal.pone.0284840 (PMC10351705; doi:10.1371/journal.pone.0284840)
Supplement: S1 Appendix — (DOCX) [file pone.0284840.s001.docx]

**Appendix A. Scale Items Used in Studies 1 and 2**

**Perceived AI ability-power fit**

(1) AI’s abilities are a good fit with the power it has been granted in this decision-making process.

(2) The match is very good between AI’s abilities and the power it has been granted in this decision-making process.

(3) AI’s abilities and expertise provide a good match with the power it has been granted in this decision-making process.

**Procedural justice**

(1) This process for making personnel decisions is fair.

(2) The way this decision-making process determines which candidates receive job opportunities seems fair.

(3) This process regarding how candiadates are evaluated is fair.

(4) The outcomes of this decision-making process are fair.

**Manipulation check of the order of decision makers**

(1) In the two interview rounds presented in the scenario above, AI interviewers (rather than humans) are the first-round interviewers.

(2) In the two interview rounds presented in the scenario above, humans (rather than AI interviewers) are the second-round interviewers.

**Manipulation check of AI ability**

(1) In this promotion decision-making process, AI could put all factors related to future performance into context when evaluating candidates.

(2) In this promotion decision-making process, AI could take into account how all factors related to future performance fit together.

(3) In this promotion decision-making process, AI could look at each factors in the light of others to foresee candidates’ future performance.
